# Cognitive Behavioural Therapy/Motivational Interviewing in Emergency Settings in Child and Adolescent Psychiatry

**DOI:** 10.1192/j.eurpsy.2025.116

**Published:** 2025-08-26

**Authors:** T. Gargot

**Affiliations:** Child and Adolescents psychiatry, University Hospital Tours, Tours, France

## Abstract

**Abstract:**

Worldwide, suicidal ideation in youth varies between 14% and 23 %. Between 5% and 16% have made a suicidal attempt. The prevalence has increased recently. However, we measure a decrease in suicide deaths (Van Meter et al., 2021). Suicide is the second cause of death after accidents. Most psychiatric disorders appear during or before adolescence (Solmi et al., 2022), which makes it a critical period to begin treatment. Development induces peculiarities like more dependence on the environment, insight difficulties, and impulsivity.

Risk factors should be assessed like previous suicidal attempt history, active suicidal ideation, trauma, alcohol misuse, and drug misuse (Pernau et al., 2024).

The management of these situations stresses several challenges. Several interventions were designed but did not show specificities on subsequent suicide prevention. However, they decreased immediate psychiatric hospitalisations, increased mental health service use, and showed mild improvement in subsequent depressive symptoms (Pitt et al., 2024).

Studies in non-specific factors and therapeutic alliance operationalized in motivational interviewing open perspectives like the importance of reflective listening, open questions, semi-directivity and shared objectives (Miller & Moyers, 2021).

Understanding of depression enlightens specific simple approaches like behavioral activation (Richard et al., 2016; Kanter et al., 2010) and problem-solving therapy (Bell & D’Zurilla, 2009).

Specific tools like joint crisis plans (Jong et al., 2016) are useful and ethical on paper or in apps (Stanley et al., 2018)

Organization of care is of paramount importance like phone and letters contact like the program Vigilans (Plancke et al., 2020).

Bibliotherapy and internet CBT tools (Linardon et al, 2024 ; Hedman-Lagerlöf, et al., 2023 ; Lewis et al., 2012) could be useful to improve mental health care accessibility with a small cost.

**Disclosure of Interest:**

None Declared

